# Introducing a new themed collection on emerging technologies for research models of human neuronal disorders *in vivo* and *in vitro*

**DOI:** 10.1042/NS20220065

**Published:** 2022-09-30

**Authors:** Thomas J. Cunningham, Clare Stanford

**Affiliations:** 1 MRC Prion Unit at UCL, Institute of Prion Diseases, University College London, London, UK; 2 Department of Neuroscience Physiology and Pharmacology, University College London, London, UK

**Keywords:** biological models, biotechnology, Editorial

## Abstract

This themed collection of articles was prompted by a collaboration between *Neuronal Signaling* and the British Neuroscience Association. The Biochemical Society and Portland Press organised a symposium at the BNA Festival of Neuroscience in 2021, focused on the development and use of experimental models of human neuronal disorders. One aspect dealt with how new technologies are being (or could be) used both as a substitute for, or to complement, research that uses whole animal models. Another aspect discussed factors that need to be considered when appraising the validity of animal models of complex, multifactorial neuronal disorders. Given its relevance to the scope of *Neuronal Signaling*, the journal’s Editorial Board developed a themed collection of content around this symposium entitled *Emerging technologies for research models of human neuronal disorders in vivo and in vitro*.

We were delighted that speakers from the symposium and other experts working in this field agreed to submit reviews for the collection, which offers an invaluable resource both for researchers who are already experts in this field and those who need merely to learn about its scope and potential.

This series of articles starts with a review by Baena-Montes et al. [1], who highlight the capacity of human induced pluripotent stem cell (iPSC) technologies to recapitulate human cellular phenotypes and α-synuclein pathologies in Parkinson’s disease. Important aspects of this approach include not only the feasibility of studying neuronal function and dysfunction *in vitro*, and its contribution to the 3Rs (Replacement) but also the ability to couple the research of a ‘disease in a dish’ with patients’ genetic background. In this review, the authors explain the background to this technology and highlight some technical challenges and appraise its potential in the context of their own research of the effects of *SNCA* gene mutations and α-synucleinopathies.

iPSC technology also has the potential to provide novel therapies, but the next article in the collection (Bartly et al. [2]) reviews research using foetal tissue transplantation to develop novel treatments for Huntingdon’s disease. The authors conclude that this approach is still warranted, given that newer iPSC technologies have not yet proven to be a superior replacement. Moreover, the authors explain how findings from foetal transplants can help to inform strategies for development of iPSC-based therapeutics.

Notwithstanding the important progress in *in vitro* technologies, effective modelling of complex *in vivo* systems requires deep understanding of diverse cellular interactions and local environment physiology. In the next review, Potjewyd et al. [3] provide an in-depth description of the cell biology and function of the blood–brain barrier (BBB). The focus of this review is how recent innovations with hydrogels and multicellular iPSC systems have provided improved, physiologically relevant 3D models for studying BBB dysfunction and associated disease states.

From a group that pioneered genetic mouse models of Down syndrome and Alzheimer’s disease, Yu et al. [4] highlight the respective limitations of mouse and cellular models. For instance, the differences in human and mouse chromosomal synteny or glial biology are discussed in detail, as are the limitations resulting from cell immaturity in human cellular models. Despite these challenges, the article explains how emerging technologies, using cellular models (such as those discussed above) can be used to help us understand and explain human-specific cellular and molecular pathologies. In short, the recent development of human-iPSC-mouse chimaeras *in vivo* may offer the best of both worlds.

The following reviews shift the emphasis of the collection to whole animal studies *in vivo*. Coup and Bossing [5] discuss how the fruit fly, which has highly tractable genetics, offers a powerful non-mammalian alternative approach to *in vivo* studies of fundamental and evolutionarily conserved neuronal signaling pathways. It is clear that research of the fruit fly has provided valuable insights into the processes of neuronal injury and regenerative repair, ranging from Wallarian degeneration to glial cell responses.

In the next review of the series, Pohl and Hörnberg [6] discuss mouse models of *de novo* mutations that affect the neuroligin gene family, the expression of which are important modulators of synaptic function. The review appraises evidence that mutations impart diverse molecular and behavioural phenotypes, and aims to consolidate converging mechanisms of neuronal signalling. In covering this fascinating field, the authors explain how the findings can help us to explain links between synaptic function and social behaviour.

The final review in the collection deals with animal models of Parkinson’s disease: ranging from genetically altered mice to non-human primates with a neurotoxic (MPTP) lesion. Lama et al. [7] acknowledge that no single animal model recapitulates Parkinson’s disease, as it is manifest in humans, but they highlight the strengths of each model for understanding different facets of the disease. An invaluable feature of this review is that it offers advice to researchers on how to decide which model is best suited to meet the objectives of the experiments in prospect. This guidance will certainly be a greatly appreciated ‘go-to’ resource for researchers, whether their research objectives are to understand the aetiology of Parkinson's disease, its pathology, pathogenesis or therapeutics, including non-motor signs of the disease.

The commissioning of these articles coincided with the emergence of COVID-19 and yet all the authors sent us their promised contributions, despite the many personal and professional challenges arising from the pandemic. We are indebted to all these distinguished authors for all their contributions to this Themed Collection and for their conscientious support.

## Open Access

Open access for this article was enabled by the participation of University College London in an all-inclusive *Read & Publish* agreement with Portland Press and the Biochemical Society under a transformative agreement with JISC.

## Abbreviations

BBB: blood–brain barrier
iPSC: induced pluripotent stem cell
MPTP: 1-methyl-4-phenyl-1,2,3,6-tetrahydropyridine
